# The N-terminus of Bunyamwera orthobunyavirus NSs protein is essential for interferon antagonism

**DOI:** 10.1099/vir.0.021774-0

**Published:** 2010-08

**Authors:** Ingeborg van Knippenberg, Charlie Carlton-Smith, Richard M. Elliott

**Affiliations:** Biomedical Sciences Research Centre, University of St Andrews, North Haugh, St Andrews, Fife KY16 9ST, UK

## Abstract

Bunyamwera virus NSs protein is involved in the inhibition of cellular transcription and the interferon (IFN) response, and it interacts with the Med8 component of Mediator. A spontaneous mutant of a recombinant NSs-deleted Bunyamwera virus (rBUNdelNSs2) was identified and characterized. This mutant virus, termed mBUNNSs22, expresses a 21 aa N-terminally truncated form of NSs. Like rBUNdelNSs2, mBUNNSs22 is attenuated in IFN-deficient cells, and to a greater extent in IFN-competent cells. Both rBUNdelNSs2 and mBUNNSs22 are potent IFN inducers and their growth can be rescued by depleting cellular IRF3. Strikingly, despite encoding an NSs protein that contains the Med8 interaction domain, mBUNNSs22 fails to block RNA polymerase II activity during infection. Overall, our data suggest that both the interaction of NSs with Med8 and a novel unidentified function of the NSs N-terminus, seem necessary for Bunyamwera virus to counteract host antiviral responses.

*Bunyamwera virus* (BUNV) is the type species of both the genus *Orthobunyavirus* and the family *Bunyaviridae*, members of which are important human pathogens causing diseases such as febrile illness and haemorrhagic fever. Bunyaviruses possess a tri-segmented, negative-sense RNA genome that encodes four structural proteins: the viral RNA-dependent RNA polymerase (L protein) on the large (L) segment, two glycoproteins (Gn and Gc) on the medium (M) segment, and the nucleoprotein (N) on the small (S) segment. In addition, orthobunyaviruses encode one or two non-structural proteins, NSm on the M segment and NSs (for most but not all orthobunyaviruses; Mohamed *et al.*, 2009) on the S segment (Nichol *et al.*, 2005; Schmaljohn & Hooper, 2001).

The NSs protein is the major viral interferon (IFN) antagonist involved in evading host innate immune responses (Bridgen *et al.*, 2001; Weber *et al.*, 2002). NSs was shown to interact with Med8 (Léonard *et al.*, 2006), a component of the Mediator complex that is involved in regulating the activity of cellular RNA polymerase II (RNAPII; Malik & Roeder, 2005). During infection with wild-type (wt) BUNV cellular RNAPII is degraded, presumably as a result of the interaction between NSs and Med8 (Léonard *et al.*, 2006). Thus, NSs is thought to antagonize the IFN response by a general block of transcription of all host genes including IFN. This general transcriptional block caused by NSs is at least partially responsible for the observed shut off of host cell protein synthesis (Bridgen *et al.*, 2001; Thomas *et al.*, 2004; Hart *et al.*, 2008).

The N and NSs proteins are translated from overlapping open reading frames (ORFs) in a single mRNA transcribed from the S segment. To examine the role of NSs in infection, a recombinant virus lacking NSs (rBUNdelNSs2) was created in which NSs expression was abrogated by changing the tandem AUG initiation codons to ACG codons and converting codon 3 (serine) to a stop codon. In addition, codons 4 and 5 were changed from leucine to proline (Bridgen *et al.*, 2001). Subsequently, a potential downstream AUG-initiation codon at position 30 was changed to ACG (Fig. 1a; Hart *et al.*, 2008). All the mutations introduced to create rBUNdelNSs2 are silent with respect to the N ORF.

During routine analysis of newly prepared viral stocks derived from isolated plaques, lysates of infected BHK cells were checked for expression of NSs by Western blotting as described previously (Blakqori *et al.*, 2009). As expected, wtBUNV expressed the 11 kDa NSs protein (Fig. 1b), but surprisingly one particular stock of the NSs-deleted virus, subsequently named mBUNNSs22, expressed a ∼9 kDa protein that cross-reacted with BUNV NSs antiserum (Fig. 1b). Analysis of the nucleotide sequence encompassing the N and NSs start codons in the S segment revealed that codon 22 in the NSs ORF was changed from GUG to AUG [nt 168–170 in the (+) sense S RNA], creating a new translation initiation codon in the NSs gene (Fig. 1a). The NSs protein resulting from initiation at this new AUG (NSs22 protein) is predicted to have a molecular mass of 8.76 kDa, which is consistent with the observed size difference compared with full-length NSs (Fig. 1b). The mutation also led to a conservative amino acid change in the N protein from arginine to histidine at residue 28 (Fig. 1a). Arginine 28 is not conserved between different orthobunyavirus N proteins and mutations at this position have only a minor impact on virus replication (Eifan & Elliott, 2009).

Treatment of infected cells with the proteasome inhibitor MG132 (10 μM) leads to accumulation and therefore more sensitive detection of NSs (Hart *et al.*, 2008). We compared NSs expression in cells infected with another rBUNdelNSs2 stock to those infected with wt- and mBUNNSs22 viruses in the presence of MG132 (Fig. 1c). Whereas the latter two lysates showed bands corresponding to full-length and truncated NSs, respectively, no NSs protein could be detected in extracts of rBUNdelNSs2-infected cells. In addition, nucleotide sequence analysis of the S segment of rBUNdelNSs2 confirmed that only the mutations that were originally introduced to create the NSs-deletion mutant were present (codons 1–5 and 30; Bridgen *et al.*, 2001; Hart *et al.*, 2008), and codon 22 remained as GUG.

Multicycle virus growth in BHK cells was analysed as described previously (Shi *et al.*, 2009) at both 33 °C, the temperature at which viral stocks are prepared, and 37 °C, the temperature used for most experimental work with BUNV. At both temperatures both mutant viruses grew to titres about 10-fold lower than wt virus (Fig. 1d, e). Interestingly, mBUNNSs22 grew to slightly lower titres than delNSs at 33 °C (4.4×10^5^ p.f.u. ml^−1^ vs 1.5×10^6^ p.f.u. ml^−1^; Fig. 1d), but slightly higher titres at 37 °C (4×10^6^ p.f.u. ml^−1^ vs 7.6×10^5^ p.f.u. ml^−1^; Fig. 1e), an observation that was consistent in repeated experiments (data not shown).

Although mBUNNSs22 was attenuated in the partially IFN-deficient BHK cell line (Habjan *et al.*, 2009), we speculated that this virus may be less sensitive to the host IFN response than rBUNdelNSs2, since it expressed a large fragment of the viral IFN antagonist. In IFN-competent A549 cells, mBUNNSs22, like rBUNdelNSs2, was severely attenuated, and grew to titres approximately 1000-fold lower than wtBUNV (Fig. 2a). The levels of IFN induction in A549 cells were analysed using the biological assay described by Mohamed *et al.* (2009). Briefly, the medium from infected A549 cells was collected at 24 h post-infection (p.i.), UV-inactivated and then used to induce protection of indicator cells from encephalomyocarditis virus (EMCV) infection. Infection by rBUNdelNSs2 or mBUNNSs22 resulted in secretion of significantly higher amounts of biologically active IFN than infection with wtBUNV (Fig. 2b), indicating that mBUNNSs22, like rBUNdelNSs2, is a strong IFN inducer. Finally, we compared the plaque phenotypes of wtBUNV, mBUNNSs22 and rBUNdelNSs2 in A549 cells and in A549-NPro cells that express the bovine viral diarrhea virus NPro protein (Hale *et al.*, 2009). NPro induces proteasome-mediated degradation of IRF-3, a cellular transcription factor essential for the production of IFN-*β* in response to virus infection (Hilton *et al.*, 2006). The cells were infected with approximately 50 p.f.u. of virus and stained after 5 days incubation at 37 °C. Only wt virus produced plaques on naïve A549 cells, but all three viruses formed plaques in A549-NPro cells (Fig. 2c). Thus, the attenuation of mBUNNSs22 in naïve A549 cells can be relieved by degradation of IRF-3, suggesting that mBUNNSs22, like rBUNdelNSs2, had lost its IFN-antagonist function.

The mechanism by which wtBUNV blocks the IFN response has been proposed to involve NSs-mediated blocking of phosphorylation of serine-2 in the heptad repeat in the RNAPII C-terminal domain (CTD; Thomas *et al.*, 2004; Léonard *et al.*, 2006). To test whether mBUNNSs22 was impaired in its ability to inhibit serine-2 phosphorylation, BHK cells were infected with wtBUNV, rBUNdelNSs or mBUNNSs22 and cell lysates analysed by Western blotting using antibodies specific for the serine-2 phosphorylated CTD of RNAPII (Ser2-P RNAPII; H5, Covance Research Products) or for RNAPII irrespective of its phosphorylation state (8WG16; Covance). As observed consistently in repeated experiments, during wtBUNV infection an increase in the signal for NSs correlated with a decrease in the signal for Ser2-P RNAPII and later also RNAPII in any phosphorylation state. Although it cannot be concluded per se that NSs is directly responsible for the degradation of RNAPII, it seems plausible that NSs disturbs serine-2 phosphorylation of the CTD and this leads to a stalled RNAPII complex, which is then targeted for degradation. Generally, no decrease in RNAPII levels was observed in rBUNdelNSs2-infected cell extracts where no NSs was expressed (Fig. 3a), confirming that NSs is responsible for RNAPII degradation. In extracts of cells infected with mBUNNSs22 a clear signal for the truncated NSs protein was detected, but no decrease in RNAPII levels could be observed (Fig. 3a). These results confirmed that mBUNNSs22 had lost the ability to block phosphorlyation or induce degradation of RNAPII and thus to counteract the host IFN response.

Previous analyses had mapped the Med8-interacting domain in NSs to residues 83–91, and showed that NSs proteins with N-terminal truncations of 10, 40 or 49 aa could still interact with Med8 (Léonard *et al.*, 2006). This implies that the truncated NSs22 protein expressed by the mutant virus would be capable of interacting with Med8, and yet no degradation of RNAPII could be detected. Therefore, the interaction between the NSs C terminus and Med8, though essential, seems not to be sufficient to block phosphorylation of CTD-Ser2 or to promote degradation of RNAPII during infection.

The ability of mBUNNSs22 to shut off host protein synthesis was compared to that of wt and rBUNdelNSs2 viruses in A549 and Vero cells (Fig. 3b) by metabolic labelling with [^35^S]methionine as described previously (Léonard *et al.*, 2006). Whereas wtBUNV caused host protein synthesis shut off in both cell types, hardly any shut off was observed for rBUNdelNSs2 or mBUNNSs22 (Fig. 3b). The shut off observed in wtBUNV-infected cells is the result of the effect of NSs on both cellular translation and transcription (Bridgen *et al.*, 2001; Hart *et al.*, 2008; Thomas *et al.*, 2004) and the blocking of RNAPII activity is a major contributing factor. Thus, the results in Fig. 3(b) correlate well with those in Figs 2 and 3(a), and are in agreement with the hypothesis that inability of NSs to block RNAPII activity prevents virus-mediated shut off of host cell gene expression and consequently leads to the induction of IFN.

Compared to the amount of N protein, the level of NSs22 protein produced by mBUNNSs22 appeared to be lower than that of full-length NSs made by wtBUNV, at least for the first 12 h of infection (Figs 1b and 3a). This difference was consistently observed in repeated Western blot experiments (not shown) and may reflect a lower or slower rate of synthesis of the truncated protein. Alternatively, it could be the result of increased instability of the mutant protein. Two observations seem to point towards the latter explanation: (i) the NSs22 protein was not consistently detected in plasmid-transfected cells (data not shown), and (ii) the C-terminally truncated NSs protein expressed by BUNNSs-T83 (Léonard *et al.*, 2006) is also difficult to detect in infected cells (unpublished observations).

The first NSs-deletion virus generated, rBUNdelNSs9a, was found to express a truncated NSs protein (NSs30) from the AUG at codon 30, but this virus had the same phenotype in BHK cells as the subsequently made rBUNdelNSs2 virus (Bridgen *et al.*, 2001; Hart *et al.*, 2008) and was a potent inducer of IFN (Weber *et al.*, 2002). The data presented here are in agreement with, and extend, those obtained for rBUNdelNSs9a. It seems unlikely that NSs30 is expressed during wtBUNV infection since most ribosomes will initiate either at the N start codon [that is in a weak ‘Kozak’ sequence (Kozak, 2002)] or at the NSs start codon (that is in a slightly stronger Kozak context). On the other hand, the start codon to initiate NSs22 translation is in a relatively strong Kozak context even though it was not originally an initiation codon. This may explain why the mutation that rescued truncated-NSs expression occurred at this site rather than reversion of codon 30.

The fact that mBUNNSs22 arose spontaneously suggests a selective pressure for some function of NSs to be regained by the virus. Our results indicate that although mBUNNSs22 expresses a large part of the viral IFN antagonist protein, this mutant virus behaves indistinguishably from rBUNdelNSs2 in its inability to inhibit IFN induction. The selective pressure must thus be for another function of NSs, which could be to inhibit protein translation (Hart *et al.*, 2008), to counteract induction of apoptosis (Kohl *et al.*, 2003) or to regulate the viral RNA polymerase (Weber *et al.*, 2001). Attempts to examine the effect of NSs22 on viral polymerase activity using the minigenome assay (Weber *et al.*, 2001) were thwarted by the instability of the truncated protein, though other means to measure this effect are currently being explored.

The results presented in Figs 2 and 3 demonstrate the requirement of the first 21 aa of the NSs protein for its IFN-antagonist function. Although the interaction between NSs and Med8 is thought to be involved in blocking host transcription (Léonard *et al.*, 2006), the presence of the interacting domain in NSs (located between aa 83 and 91; Léonard *et al.*, 2006) is apparently in itself not enough to lead to the blocking of RNAPII activity. This suggests that the interaction with Med8 alone is not sufficient to inhibit host transcription and that other factors requiring the NSs N terminus are involved in this mechanism. The identity of these factors and the mechanism of RNAPII inhibition are currently under investigation.

## Figures and Tables

**Fig. 1.**
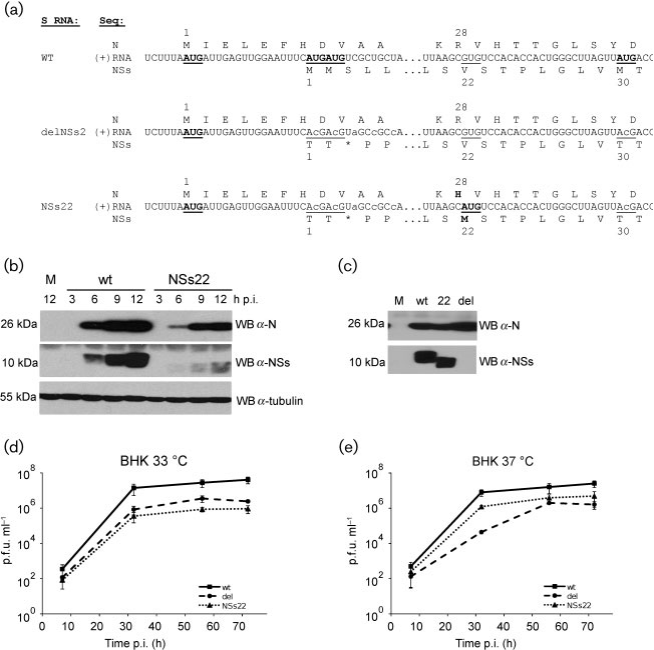
Characterization of a mutant Bunyamwera virus that expresses an N-terminally truncated NSs protein. (a) Schematic of S segment RNA sequences surrounding the N and NSs ORF start codons for wtBUNV, rBUNdelNSs2 (delNSs2) and mBUNNSs22 (NSs22). Shown are nt 83–119 and 162–197 of the S (+) RNA. The numbering of the relevant codons is indicated, AUG start codons are underlined and bold, and an asterisk (*) marks a stop codon. Amino acid sequences for N and NSs are shown above and below the RNA sequences, respectively. (b) Western blot analysis of BHK cells infected with wtBUNV (wt) or mBUNNSs22 (NSs22) or mock-infected (M). Strips of the blot were probed with the antibodies indicated on the right; size markers are indicated on the left. (c) Western blot analysis of BHK cells infected with wtBUNV, mBUNNSs22 (22), rBUNdelNSs2 (del) or mock-infected. MG132 (10 μM) was added to the medium at 5 h p.i. and cell extracts were prepared at 12 h p.i. (d, e) Multi-step growth curves of wtBUNV, rBUNdelNSs2 and mBUNNSs22 in BHK cells at 33 °C (d) and 37 °C (e). Shown are mean values of triplicate infections.

**Fig. 2.**
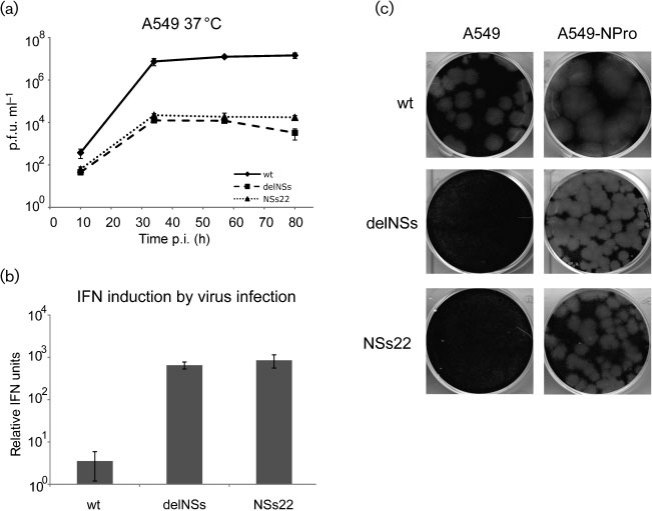
mBUNNSs22 is attenuated in IFN-competent cells and is a potent IFN inducer. (a) Multi-step growth curves of wtBUNV, rBUNdelNSs2 and mBUNNSs22 virus in A549 cells. Shown are mean values of triplicate infections. (b) Levels of IFN induced in A549 cells after 24 h infection with wtBUNV, rBUNdelNSs2 or mBUNNSs22. The relative IFN content of medium from infected cells was measured by comparing the dilution that could protect indicator cells from EMCV infection. (c) Plaque formation in IFN-competent A549 cells (left panels) and IFN-deficient A549-NPro cells (right panels). Cells were infected with wtBUNV, rBUNdelNSs2 or mBUNNSs22 as indicated and were stained for plaque formation after 5 days incubation at 37 °C.

**Fig. 3.**
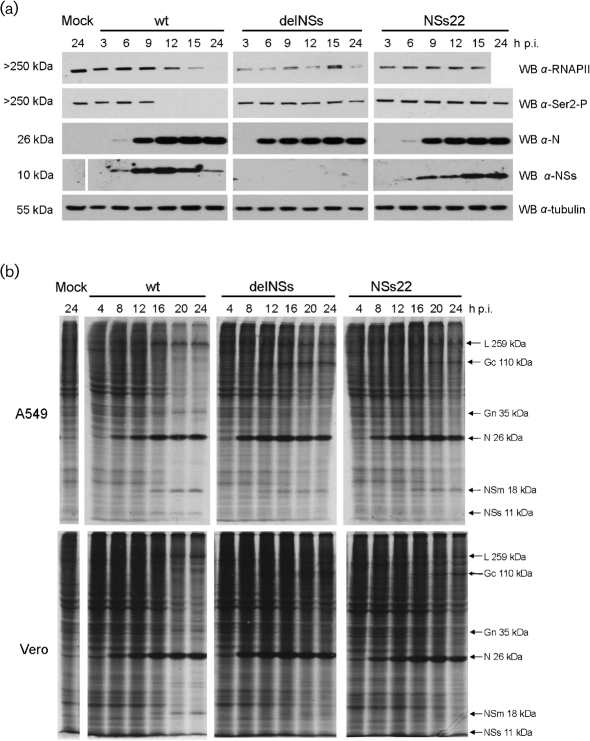
mBUNNSs22 does not degrade RNAPII or cause shut off of host protein synthesis. (a) Western blot analysis of BHK cells infected with wtBUNV, rBUNdelNSs2, mBUNNS22 or mock-infected, and harvested at the indicated times p.i. Size markers are indicated on the left, and antibodies used on the right. *α*-RNAPII, antibody against RNAPII-CTD, regardless of its phosphorylation state; *α*-Ser2-P, antibody specific for serine-2-phosphorylated CTD of RNAPII. (b) Metabolic labelling of infected cells. A549 cells (top panel) and Vero cells (bottom panel) were infected with wtBUNV, rBUNdelNSs2, mBUNNSs22 or were mock-infected. Cells were labelled with [^35^S]methionine for 1 h prior to the indicated time p.i., and cell lysates were analysed by SDS-PAGE. Viral proteins and their sizes are indicated on the right.
